# Vascularized Palmaris Longus Tendon Graft in Delayed Tendon Injuries of Hand

**DOI:** 10.1055/s-0044-1792128

**Published:** 2024-11-19

**Authors:** Yog Raj Handoo

**Affiliations:** 1 Department of Plastic Surgery, RD Plastic Surgery Center, New Delhi, India

**Keywords:** vascularized tendon graft, vascularized palmaris tendon graft

## Abstract

Delayed tendon injuries of the hand often require auto tendon grafts, the success depending largely on tendon type, its own synovial cover or provided by tendon bed, and tendon bed vascularity. Tendon graft healing has been extensively studied, where tendon does increase its vascular response to trauma, attrition, or degeneration. Initially surviving through synovial fluid imbibition, followed by vascular invasion from surrounding tissues. Tendon grafts without synovial covering or in hypovascular/scarred beds have increased vascular response in the form of flimsy tissue which vascularises tendon grafts but later, they become adhesions, restricting tendon movement and final functional finger results. To overcome this hypovascular state, tendon grafts are substituted with vascularized tendon grafts. We present two cases of vascularized palmaris tendon graft in delayed tendon injuries of hand having hypovascular tendon beds, along with operative technique and results along discussion and conclusion.

## Introduction

Presentation of delayed tendon injuries of hand is a common occurrence, necessitating repair by tendon graft, usually autograft. While results of primary tendon surgery are good, 1 functional results of delayed tendon graft depend lot on donor tendon, tendon bed, and its vascularity. 2 Tendon graft healing has been studied extensively, be it animal models 3 or clinical experience. 4 5 Normally, tendon blood supply comes from “osteotendinous junction, musculotendinous junction, and synovial sheath through meso-synovium.” 6 Tendons are hypovascular structures, yet mount “vascular donor response by increased vascularity to trauma, attrition, or degeneration.” 7 8 Tendon grafts initially survive by imbibition of synovial fluid, 9 when covered by synovium, but if not having synovial layers, then by vascular flimsy tissue invasion from surrounding tissues and also increased vascularity from two ends of tendon graft anastomosis. 10 Depending upon the thickness of the tendon, while being vascularized, areas of necrosis may occur which heal by fibrosis and adhesion to surrounding structures. 11 Finally, flimsy vascular connections between tendon bed and revascularized tendon graft become fibrosed and hence are adhesions of repaired tendon, resulting in restricted tendon mobility and ultimately less than satisfactory results of tendon surgery. Scarred tendon bed, whether due to trauma, infection, or degeneration, results in thick adhesions because of thicker vascular invasion from tendon bed.

To prevent excessive vascular invasion from surrounding tissue, the endovascularity of the tendon is increased either by vascular tissue wrap in continuity to tendon graft or by microvascular anastomosis to adjacent donor vessels. Such vascularized tendon grafts have less vascular invasion from surrounding tissues, compared with nonvascular tendon grafts, and better results. 12 Palmaris longus tendon is the most common tendon being used for tendon graft in hand injuries. 13 It has multiple vascular pedicles arising from the ulnar artery. Palmaris longus tendon graft is vascularized by anastomosis of its vascular pedicle to donor blood vessels of the palm of hand as free vascularized tendon graft.

## Technique

**Fig. 1:**
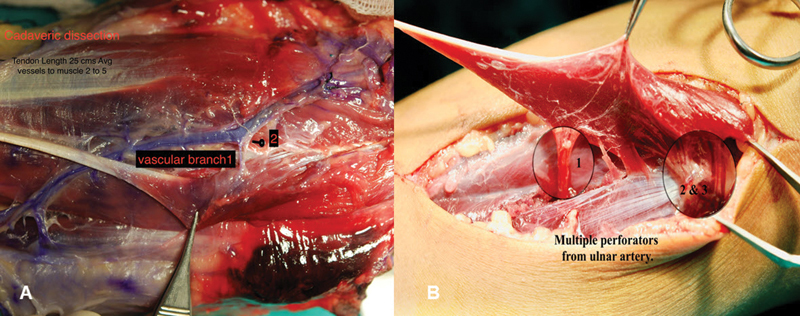
( **A** ) Palmaris longus muscle tendon and two vascular pedicles in cadaveric dissection. ( **B** ) Three vascular pedicles during surgery, magnified for better visualization.

To familiarize with vascular pedicles of palmaris longus muscle, methylene blue was injected in the brachial artery of a single fresh cadaveric arm and dissection of the palmaris longus muscle and tendon with synovium was done. Three to four small vessels were supplying muscle from the ulnar artery ( Fig. 1A ). One pedicle near the muscle origin and one near the musculotendinous junction were considered of adequate size for vascular anastomosis ( Fig. 1B ).

Both flexor digitorum profundus (FDP) and flexor digitorum superficialis (FDS) tendons in their fibrous sheath are exposed from the tip of the finger to the palm by Brunner's incision (zigzag incision) and fibrous sheath, pulleys, synovial lining, condition, and adhesion of residual tendons are noted. The tendon bed is prepared and the decision to use a tip-to-palm tendon graft is confirmed. The synovial lining of the fibrous sheath is checked again. All residual tendons, both FDP and FDS, are removed, preserving more than 1.5 cm of the distal insertion of FDP and FDS tendon insertion, and excising the rest of the chiasma as well. A feeding tube (size 6) is gently passed through the fibrous sheath as a guide tube and to check the adequacy of the pulleys and determine the length of the tendon graft needed from the tip of the finger to the palm. The harvested tendon is then laid on the palm and the layout of the pedicle is determined. The recipient artery and vein are identified ( Fig. 1B ), avoiding digital vessels, which may have been potentially compromised due to previous injury or infections. Instead, muscular vessels to interossei muscles or skin perforators are chosen for anastomosis.

**Fig. 2:**
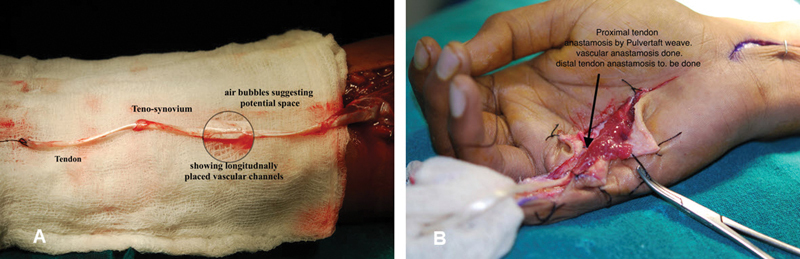
( **A** ) Palmaris longus muscle tendon along with tenosynovium, dissected out with vascular pedicles in continuity, being perfused by its own pedicle ensuring perfusion before being disconnected for transfer to hand. ( **B** ) Tendon along with sleeve of muscle transferred to hand.

**Fig. 3:**
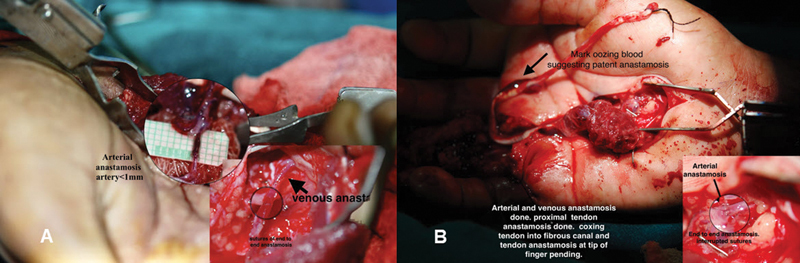
( **A** ) End-to-end arterial anastomosis for artery and for vein for case no 1 and ( **B** ) same for case no 2. Also, note some blood oozing after successful arterial and venous anastomosis.

Proximal tendon-to-tendon anastomosis is done using the Pulvertaft weave method, protecting both donor and recipient vessels ( Fig. 2 ). Before that, a dry run of the tendon in the fibrous sheath is performed. Once the final position of the donor vessels is determined, the distal part of the tendon is withdrawn into the palm. Vascular anastomosis is done with extra laxity of vessels to ensure a relaxed lie ( Fig. 3 ). After confirming patency for 15 minutes, the tendon is coaxed into the fibrous space and brought out to the tip. Distal tendon anastomosis to the stump of the FDP is completed. Tendon movement is rechecked to ensure the vascular anastomosis is not impeded. Skin closure is performed and Plaster of Paris (POP) splintage done. A point on the palm is chosen to check the patency of the anastomosis with a Doppler signal, unimpeded by signals from other vessels, and for postoperative patency follow-up. The skin is closed, and the final tension is rechecked. A POP slab is applied with a window for Doppler checkup. Postoperative care includes passive flexion of fingers for 1 week, followed by passive flexion and active extension for 3 weeks, then shifting to active flexion and active extension for a further 6 weeks. Splintage is continued for 3 months postexercise, with no weight bearing for 6 months.

### Case No 1



**Video 1** Good flexion of vascularized tendon grafted finger. Distal interphalangeal (DIP) joint persisting. Preop stiffness of DIP joint persisting.


**Video 2** Good power in finger against resistance applied.


**Fig. 4:**
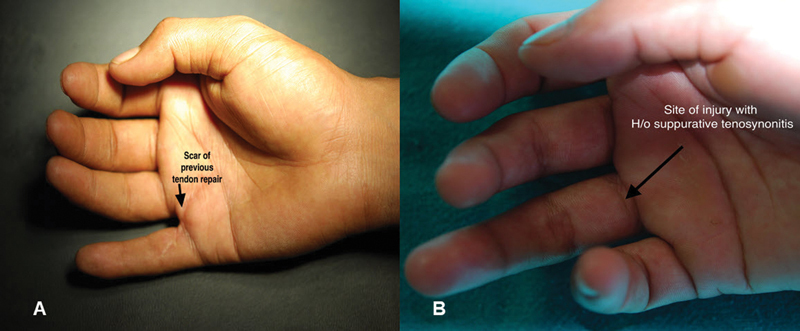
( **A** ) Case no 1 having failed primary surgery for tendon injury and ( **B** ) case no 2 with site of injury and had treated suppurative tenosynovitis.

A 23-year-old male sustained sharp cut in the 5th finger of the right hand which was operated elsewhere ( Fig. 4A ), presented to us after few months. On presentation, zone II injury with some stiffness in the distal interphalangeal (DIP) joint was noticed. Initially, the 5th finger was explored by zigzag incision and tip-to-palm graft planned. Pulleys in fibrous sheath were examined and retained. Dense adhesions of the ruptured tendon were present in the fibrous sheath. Decision to use vascularized tendon palmaris longus muscle was made and tendons were harvested along with its paratenon. Vascular supply to the palmaris longus muscle was explored and dissected out. Matching size of recipient perforator and its length was dissected in the palm of the hand sparing the digital vessels. The size of the artery usually is less than 1 mm. In the palm, anastomosis is done end-to-end both for arteries and veins. For postoperative monitoring of anastomotic patency, point on the hand for Doppler study was marked. After 3 weeks, we did color Doppler which confirmed the patent vessel. Tendon repair had good strength and glide after 6 months of surgery. Videos 1 and 2 show that the stiffness of the DIP joint persisted.

### Case No 2



**Video 3** Good flexion and extension in vascularized tendon graft finger after follow-up of 6 months.


**Fig. 5:**
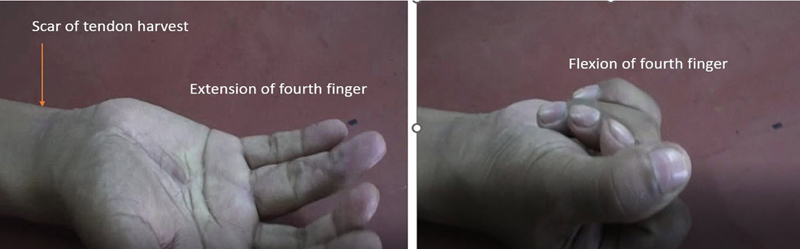
( **A** ) Final result after follow-up of 6-month period with full flexion and ( **B** ) extension in finger with vascularized tendon graft.

A 35-year-old male with FDP/FDS injury at the 4th finger ( Fig. 4B ), which had got infected. We operated after 3 months of subsidence of infection. On exploration, the fibrous sheath and synovial sheath had collapsed and scarred, without smoothness and softness. Residual tendons were scarred, gray, and adherent to the fibrous sheath. Residual tendons were excised. Pulleys were found to be adequate after gentle dilatation by metal dilators, and had absence of synovial lining. Harvested palmaris longus muscle tendon had vessels of finer size, and end-to-end anastomosis for arteries and veins was done successfully. Acoustic Doppler in immediate postoperative period and color Doppler after 3 months suggested patency of anastomosis. Functional recovery was good ( Fig. 5 ) with no stiffness of interphalangeal joints after 6 months postop ( Video 3 ).

## Discussion

Despite good results of primary tendon repair in tendon injuries, need for tendon grafts for delayed presentation of hand injuries are common. Combined results of tendon injury as analyzed by Buck–Gramcko score was 32% very good, 26.4% good, 15.1% satisfactory, and 26.5% poor. 14 To improve upon these results, research in animal experiments demonstrated significant improvement in decreasing adhesions and ultimate functional improvement by vascularizing the tendon graft by either having deep fascia in continuity to tendon 15 or using vascularized tendon based on perfusion artery as flap. Vermeylen and Monballiu in 1991 presented vascularized extensor indicis proprius (EIP) tendon graft based on the second metacarpal artery for flexor tendon graft 16 or tendon being vascularized on available vascular artery as proposed by Morrison and Cleland. 17 Tendon graft adhesion is the main cause of poor results. Vascularization of graft tendon is to increase its endovascularity and to make it less dependent on flimsy vascular invasion which ultimately leads to dense tendon adhesion. This procedure also supplements/replaces initial phase of nutrition by synovial imbibition, simultaneously transferring gliding surface of tenosynovium. Vascular channels in synovial sheath run in a longitudinal axis from which multiple anastomosing vessels are branched, producing synovial fluid for nourishing tendons and producing frictionless bed at the same time. Vessels of tendon and synovial sheath of graft get anastomosed to osteotendinous and proximal tendon stump to complete the vascular arcade.

Multiple authors have presented advantages of vascularized tendon graft in improving functional results both in scarred tendon beds or when using extrasynovial tendon as donor graft. Moriyama 15 in 1992 presented a comparative study in 40 rabbit's legs, where gliding tendon beds were destroyed by liquid nitrogen, and randomly vascularized and nonvascularized tendon grafts transferred. Histologically vascularized tendons fared better with less adhesions to surrounding tissues. Singer et al 18 used primates where seven vascularized extensor hallucis longus tendon grafts were compared with eight nonvascularized tendon grafts placed in fibro-osseous canals. After 5 months, those with vascularized tendon grafts had patent vascular pedicles and fared better in terms of simulated total active motion of toes and rupture rate.

In clinical series, Cavadas et al 19 in 2006 reported a single case of tendon reconstruction of ring finger using FDS of the same finger as free vascularized tendon from branch arising from the ulnar artery and anastomoses to the palmar arch. Ulnar artery was reanastomosed. Functional result reported was good. Cavadas et al 20 again in 2015 presented a review of 38 patients with 40 flexor tendon reconstructions where 37 pedicled flaps were used and 3 free flaps were used. While for single-stage flexor tendon repair, vascularized tendon graft did better but to find a place in reconstructive armamentarium, more is needed to be done, and Cavadas in 2023 reported five cases of flexor pollicis longus (FPL) tendon repair on the branch of ulnar artery showing refinement in microsurgery where the ulnar artery did not need to be injured or resected or reconstructed. 20 Durand et al 12 and Vermeylen and Monballiu proposed graphics for vascularized EIP tendon graft based on the branch of metacarpal artery while using vascularized extensor tendon for FPL reconstruction with good result ( Table 1 ). Noteworthy is that in both animal studies and clinical studies have very few numbers of cases suggesting this to be an evolving scenario but if we consider usage of vascular tendons in cruciate ligament and Achilles ligaments, consensus is evolving that vascular tendons give better results than nonvascular tendons.

**Table 1:** Clinical applications of vascularized tendon and outcome

Zheng et al 14	2003	Vascularized plantaris tendon composite flap with skin and/or fascia in electric burns, 7 cases	Good outcome
Cavadas 20	2015	Reconstruction of FPL tendon with vascularized tendon with ulnar artery preservation	Moderate outcome
Durand et al 12	2021	Reconstruction of FPL and extensor tendon using pedicled extensor indicis propius tendon, 2 cases	Good outcome
Leversedge 6	2000	Reconstruction of FPL tendon with vascularized pedicled FDS tendon branch of ulnar artery, 5 cases	Good outcome

Abbreviations: FDS, flexor digitorum superficialis; FPL, flexor pollicis longus.

We have successfully presented the feasibility of using vascularized palmaris longus tendon without injuring the ulnar artery as vascularized tendon graft in two patients. Both vascularized tendon grafts had patent anastomosis as confirmed by acoustic Doppler and later by color Doppler, confirming that palmaris longus continued to have vascularity from anastomosed vessels. Functionally, both patients had good outcome.

## Conclusion

Vascularized tendon grafts are still in the preliminary stage and improved results are being reported both in animal experiments and clinical situations. As the microsurgical expertise improves, so shall difficult tendon, soft tissue bone injury repair employs vascularized tendons as standalone or as part of skin and fascia replacement in hand injuries.
